# Visceral fat mass determination in rodent: validation of dual-energy x-ray absorptiometry and anthropometric techniques in fat and lean rats

**DOI:** 10.1186/1476-511X-9-140

**Published:** 2010-12-09

**Authors:** Maude Gerbaix, Lore Metz, Emeline Ringot, Daniel Courteix

**Affiliations:** 1 Laboratoire de Biologie des APS, EA 3533, PRES Clermont Université, Université Blaise Pascal, 24 avenue des Landais, BP 80026, 63177 Aubière Cedex, France

## Abstract

**Background:**

Because abdominal obesity is predisposed to various metabolic disorders, it is of major importance to assess and track the changes with time of this specific fat mass. The main issue for clinicians or researchers is to use techniques for assessing abdominal fat deposition and its accumulation or changes over time, without sacrificing of experimental subjects. In the rat, techniques to investigate in-vivo visceral fat mass are lacking. The purpose of the study was to validate indirect Dual-energy X-ray Absorptiometry technique and abdominal circumference measurement as tools to predict visceral adipose tissue in rats.

Forty-three Wistar male rats from different body weight, fat mass and ages were included in the study. Visceral fat mass was assessed by weighing the total perirenal and peri-epididymal adipose tissues after dissection. Statistical methods were used to discriminate the best region of interest allowing the in-vivo measure of Central Fat Mass by DXA. Abdominal circumference was measured at the same time as the DXA scan.

**Results:**

A region of interest including Central Fat Mass from the whole body DXA scan (extending from L2 to L5 vertebrae), correlated strongly with *ex-vivo *Fat Mass (r = 0.94, p < 0.001). Abdominal circumference correlated significantly with *ex-vivo *Fat Mass (r = 0.82, p < 0.001) and Central Fat Mass (0.90, p < 0.001) in the whole group of rats. When dividing the whole group into lean and fat rats, correlations remained significant between Central Fat Mass and *ex-vivo *Fat Mass but disappeared for the lean group between abdominal circumference and *ex-vivo *Fat Mass.

**Conclusions:**

This study validates the Central Fat Mass determined by DXA as a non-sacrificial technique to assess visceral fat for in-vivo investigations in rats. The abdominal circumference measure appears useful in studying overweight or obese rats. These two techniques could be convenient tools in follow-up and longitudinal studies.

## Background

Ageing, characterized by an increase of lipid consumption and sedentary lifestyle is known to be linked with metabolic pathologies. The excess of lipid provided by food is stored in localized sites (abdomen, liver, hips) and becomes pathological, disturbing a great number of metabolic functions and contributing to the rise in metabolic syndrome [1]. Abdominal obesity is also strongly linked with diabetes mellitus and an increase of cardiovascular disease, resulting in a prevalence of adverse cardiovascular events [2,3].

The mechanisms leading to the local accumulation of adipose tissue are not fully elucidated in both humans and animals. Therefore, the main issue for clinicians or researchers is to have techniques for assessing the abdominal fat deposition and its accumulation or change over time. Moreover, the sacrifice of experimental animals permits the determination of the exact value of visceral fat tissue but prohibits follow up of the change to this tissue in vivo.

Studies on the accumulation of abdominal fat mass in animals could help to better understand the mechanisms of metabolic syndrome development but the techniques of investigation are lacking.

In rodents, the total body weight measure does not give relevant information about body composition, which is an essential parameter in this kind of investigation. On the other hand, waist circumference measures have been shown to significantly correlate with cardiovascular disease in humans [4]. Among several anthropometric measurements, waist circumference was also found to be the best predictor of intra-abdominal fat thickness in normal subjects and therefore of central obesity [5]. While the ability to predict visceral fat from circumferences and diameters in obese humans has been validated [6], no anthropometric technique has been developed in animals to date. As aforementioned, the accumulation of intra-abdominal fat can be measured by direct weighing of fat after dissection. Some studies weighed intra-abdominal visceral fat including retroperitoneal, epididymal, and mesenteric fat tissues [7-11]. Lac et al. [12] measured visceral fat mass by weighing the left perirenal fat pad alone, considering that it was representative of whole abdominal fat tissue. However, because of the sacrifice involved by such techniques, longitudinal studies are excluded. The use of computed tomography in assessing body composition has been shown to be an useful technique to quantify fat tissue deposits [7]. Magnetic resonance imaging has also been used to measure abdominal fat, and discriminate between locations of adipose tissue [13]. Unfortunately, these two techniques are hampered by availability and cost; resulting in infrequent use in longitudinal studies. Thus, no technique is currently available to estimate in vivo abdominal fat mass in animals.

Due to its excellent reproducibility [14], Dual-energy X-ray absorptiometry (DXA) has become one of the best technique for assessing body composition in humans [15] and rodents [16]. The DXA analysis software allows specific regions to be identified making it an expedient and cost-effective method to estimate abdominal adipose tissue in humans [17,18]. The validity and reliability of DXA to measure adiposity in the abdominal region has been determined in humans [19]. Kamel et al. [20] showed that central fat mass determined by this technique was strongly associated with the value of abdominal visceral fat in both men and women, but no study has investigated abdominal fat mass in rats. Similarly, abdominal circumference measures are among established criteria for human metabolic syndrome diagnosis, yet no anthropometric technique has been developed in animals.

Therefore, the purpose of the present study was to validate a DXA technique and abdominal circumference measurement as predictors of visceral adipose tissue in lean and fat male rats.

## Methods

### Animal Care

All experimental designs and procedures were made in accordance to the current legislation on animal experience in France and were approved by the ethical committee for animal experimentation (CREEA Auvergne, CE1-09). Considering a minimum of 10% difference of visceral fat mass between fat and lean rats, the statistical power calculation resulted in a number of 12 rats per groups for p < 0.05 and a type II error of 0.01%. Therefore, we included groups of 14 rats.

Forty-three Wistar male rats (CERJ Janvier^®^, Le Genest Saint-Isle, France) were included in the study. In order to measure a large range of subjects, we chose a group of 29 rats aged 11 months and 14 rats aged 7 months with a range of body weights. Among the group of 29 rats, 14 rats were previously fed with a normal diet (lean group) and 15 rats were given a high fat/high sucrose diet (fat group) for four months to increase fat mass storage. The 7-month old rats were fed with a normal diet. Rats were individually housed in a temperature-controlled room (20-22°C) and a reversed light-dark cycle (light on 20h00-08h00) was maintained. The rats had free access to water. All measures were performed in the early morning to avoid diurnal complications.

### Total Body Composition by DXA

**Figure 1:**
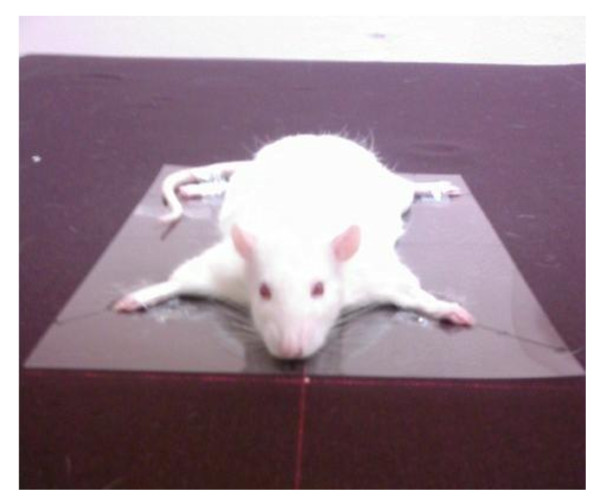
**Ventral position of rats on DXA**.

A Hologic QDR 4500 device was used with an internal adapted collimator for small animal measurements (Hologic QDR Software for Windows XP version, Copyright^© ^1986-2002 Hologic Inc.). Rat whole body measurement required 240 seconds and provided global and regional body composition results. The scan field was adjustable to a maximum of 36 cm (L) * 18 cm (W). Spatial resolution was approximately 1 mm. A special designed small animal step phantom was scanned daily to calibrate the body composition results. Rats were anaesthetized before measurements. Anesthesia consisted on an intra-peritoneal injection of a solution of Acepromazine Vetranquil^® ^(0.5 ml.kg^-1 ^of body weight) and Ketamine Imalgène^® ^(0.75 ml.kg^-1 ^of body weight). After anesthesia, rats were positioned ventrally on a reference film to reproduce the position illustrated in figure 1.

One week before sacrifice, body mass, fat mass and lean mass of all animals were assessed by DXA using specific small animal body composition software (line spacing: 0.254 mm, resolution: 0.127 mm). The coefficients of variation (CV's) were determined for these parameters from six repeated measurements with repositioning on eight animals. The CV's were 4.79%, and 0.19 % for fat mass, and body mass, respectively.

### Dissection of rats

Rats were fasted for 12 hours before sacrifice. They were euthanized by decapitation under isoflurane anesthesia. Visceral fat mass was assessed by weighing the total perirenal and peri-epididymal adipose tissues. The weights of these two tissues were combined to form the *ex-vivo Fat Mass*.

### Central fat mass (CFM) Area Determination

**Figure 2:**
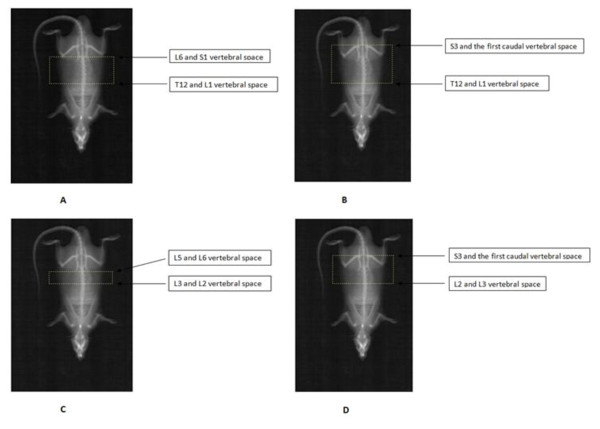
**Print-out of Dual-x-Ray Absorptiometry (DXA) scan, showing regions of interest (ROI)**. (A) ROI extending from L1 to L6 vertebrae. Fat mass from this ROI was called FM_L1_L6. (B) ROI extending from L1 to S3 vertebrae. Fat mass from this ROI was called FM_L1_S3 (C) ROI extending from L2 to L5 vertebrae. Fat mass from this ROI was called FM_L2_L5 (D) ROI extending from L3 to S3 vertebrae. Fat mass from this ROI was called FM_L3_S3.

The area determination was performed in a group of 28 rats including 14 rats aged of 7 months and the fat group (n = 14, 11 months old). In order to select the more accurate method for assessing abdominal fat mass by DXA, four regions of interest (ROI) extracted from the whole body scan were tested (figures 2A, B, C and 2D). These ROIs were defined after enlarging the total spine. They consisted of rectangular boxes extending vertically from one vertebral space to another and the lateral borders extending to the edge of the abdominal soft tissue. All trunk tissues, including intra abdominal fat and peripheral subcutaneous fat within these standardized height regions were selected for analysis. From these ROIs, we selected the region displaying the best correlation with *ex-vivo *Fat Mass. We called the fat mass measured within the chosen region "Central Fat Mass" (CFM) in order to distinguish it from the anatomic visceral fat. All investigations were performed by the same technician in order to avoid any bias linked to the inter-operator error.

### Abdominal Circumference (AC)

The abdominal circumference was performed in a group of 29 rats including the group of lean rats (n = 15) and the group of fat rats (n = 14) Before each DXA measures, abdominal circumference (AC) was assessed on the largest zone of the rat abdomen using a plastic non extensible measuring tape (Rollfix, Hoechstmass^®^, Germany) with an accuracy of 0.1 cm. Rats were placed in ventral position.

### Statistical Analysis

The Gaussian distribution for each parameter was assessed by a Shapiro-Wilk test. In case of non-normal distribution, the data were log-transformed for analyses. In order to assess the potential relationships between the variables, we performed a correlation matrix between fat mass from each DXA ROI and *ex-vivo *Fat Mass. The CV for AC measures was determined following three analyses on 13 rats. The same operator repositioned the measuring tape three times. For assessing the CV of CFM, the ROI was repositioned two times on twelve scans by the same operator. Correlation matrix was generated for AC, *ex-vivo *Fat Mass and CFM. In case of significant correlation, linear regressions were used to analyze the relationships between parameters. Characteristics differences between lean and fat rats were tested using unpaired t-tests. A correlation matrix between AC, *ex-vivo *Fat Mass and CFM distinguishing fat and lean groups was compiled. A paired Student t-test was used to compare *ex-vivo *Fat Mass and CFM for the whole population. A 2 × 2 ANOVA allowed comparing the differences between lean and fat groups, and the *ex-vivo *Fat Mass and CFM values. Analysis was carried out using SPSS Advanced Statistics software version 17 (SPSS Inc., Chicago, IL) and data are presented as mean ± SD.

## Results

**Figure 3:**
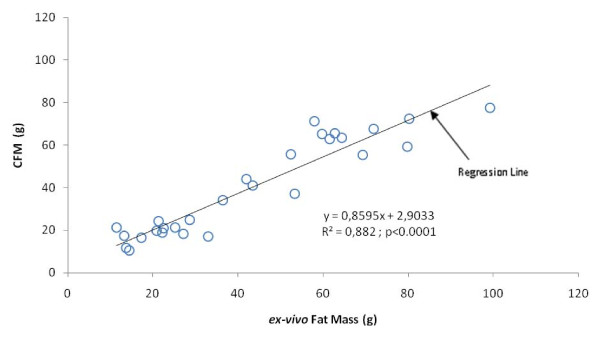
**Linear regression between CFM and *ex-vivo *Fat Mass**. CFM = Central Fat Mass; n = 28.

The characteristics of the rats involved in the CFM determination are presented table 1. Table 2 shows the correlation matrix between the four different ROIs and *ex-vivo *Fat Mass. All ROIs were significantly correlated with *ex-vivo *Fat Mass (p < 0.001) The ROI with the strongest correlation (r = 0.94, p < 0.001) was the ROI extending from L2 to L5 vertebrae (FM_L2_L5). This ROI was subsequently selected to represent the CFM. Using this ROI, the coefficient of variation calculated for CFM was 1.2%. Figure 3 displays the linear regression between CFM and *ex-vivo *Fat Mass. The explained variance for this relationship (R² = 0.88, p < 0.0001), highlights the very strong association between these data.

**Table 1:** Characteristics of the rats

	Mean	SD	Min	Max
Age (months)	9	2	7	11

Total body weight (g)	612,62	130,08	445,09	902,47

Total body Fat (g)	171,02	90,44	63,11	393,84

P Fat (%)	26,29	8,98	11,63	43,64

*ex-vivo *Fat Mass (g)	39,9	22,45	10,43	77,5

n = 28

**Table 2:** Correlation matrix for Fat Mass from the DXA ROI's and ex-vivo Fat Mass

	FM_L1_L6 (g)		FM_L1_S3 (g)		FM_L2_L5 (g)		FM_L3_S3 (g)		*ex-vivo *Fat Mass (g)	
	
	Pearson	**Sig**.	Pearson	**Sig**.	Pearson	**Sig**.	Pearson	**Sig**.	Pearson	**Sig**.
FM_L1_L6 (g)	1,00									

FM_L1_S1 (g)	0,808**	0,001	1,00							

FM_L2_L5 (g)	0,850**	0,001	0,954**	0,001	1,00					

FM_L3_S3 (g)	0,823**	0,001	0,971**	0,001	0,983**	0,001	1,00			

*ex-vivo *Fat Mass (g)	0,835**	0,001	0,933**	0,001	0,940**	0,001	0,928**	0,001	1,00	0,001

n = 28; ** p < 0.01

**Figure 4:**
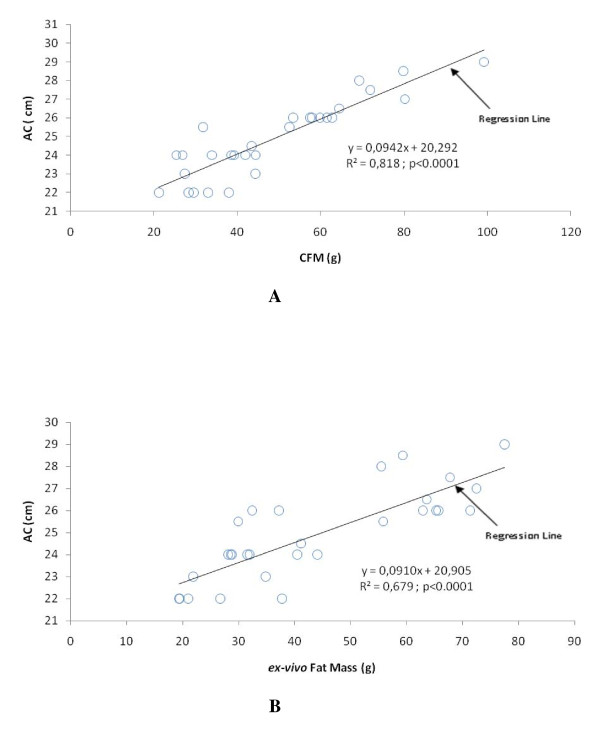
**Linear regressions for Abdominal Circumference**. (A) Linear regression between Abdominal Circumference and CMF by DXA. CFM = Central Fat Mass; AC = Abdominal Circumference; n = 29. (B) Linear regression between Abdominal Circumference and ***ex-vivo ***Fat Mass. AC = Abdominal Circumference; n = 29

The characteristics of lean, fat and the whole group rats are presented in table 3. As expected, the characteristics of fat rats were significantly larger than those of lean rats. The CFM displayed higher values than *ex-vivo *Fat Mass in the whole group as well as in fat and lean groups (p < 0.005). The CV for AC was 2.6 %. The correlation matrix between AC, CFM and *ex-vivo *Fat Mass for the total population (fat and lean rats) is displayed table 4. Within the whole population, AC correlated positively with both CFM (r = 0.90, p < 0.001) and *ex-vivo *Fat Mass (r = 0.82, p < 0.001). The slope of regression between AC and CFM (Figure 4A) displayed a strong association (R² = 0.82, p < 0.0001). Figure 4B shows a strong relationship between AC and *ex-vivo *Fat Mass (R² = 0.68, p < 0.0001). The correlation matrix between AC, CFM, and *ex-vivo *Fat Mass for fat and lean rats is displayed table 5. Correlation between CFM and *ex-vivo *Fat Mass remained significant in the two subgroups of rats (r = 0.60, p < 0.05 for lean rats and r = 0.734, p < 0.01 for fat rats) When considering the lean rats alone, the correlation between AC and both CFM (r = 0.51) and *ex-vivo *Fat Mass (r = 0.43) failed to reach significance. Nevertheless, for fat rats, AC correlated strongly with CFM (r = 0.93, p < 0.001) and the coefficient of correlation between AC and *ex-vivo *Fat Mass was weaker than for the whole population but remained significant (r = 0.583, p = 0.029).

**Table 3:** Characteristics of the total, fat and lean groups of rats.

	total n = 29		lean n = 15		fat n = 14		
	
	Mean ± SD	Range	Mean ± SD	Range	Mean ± SD	Range	p^a^
Age (months)	11	11-11	11	11-11	11	11-11	NS

Total body weight (g)	676,63 ± 82,35	521,86-902,47	627,06 ± 62,80	521,86-744,44	729,73 ± 647,64	647,64-902,47	0.001

Total body Fat (g)	202,03 ± 66,80	103,96-393,84	159,01 ± 39,16	103,96-248,47	248,12 ± 59,47	1170,75-393,84	0.001

P Fat (%)	29,36 ± 6,83	19,30-43,64	25,33 ± 5,52	19,30-35,63	33,68 ± 5,38	24,76-43,64	0.001

*ex-vivo *Fat Mass (g)	43,87 ± 18,40	19,35-77,5	28,86 ± 6,41	19,35-40,47	59,96 ± 12,08	59,96-12,08	0.001

CFM (g)	48,88 ± 19,44^b^	21,2-99,2	34,63 ± 9,32 ^b^	21,2-57,5	64,14 ± 15,35 ^b^	41,9-92,2	0.001

AC (cm)	24,90 ± 2,04 (SE = 0.38)	22-29	23,43 ± 1,29 (SE = 0.33)	22-26	26,46 ± 1,42 (SE = 0.38)	24-29	0.001

CFM = Central Fat Mass; AC = Abdominal circumference; SE = Standard Error; ^a ^Comparison by unpaired t-test between fat rats (n = 14) and lean rats (n = 15); ^b ^CMF significantly different of *ex-vivo *Fat Mass (paired t-test. p < 0.05); n = 29

**Table 4:** Correlation matrix for Abdominal Circumference, CFM and ex-vivo Fat Mass in total population.

	CFM (g)		AC (cm)		*ex-vivo *Fat Mass (g)	
	
	Pearson	**Sig**.	Pearson	**Sig**.	Pearson	**Sig**.
CFM (g)	1					

AC (cm)	0,899**	0,001	1			

*ex-vivo *Fat Mass (g)	0,890**	0,001	0,822**	0,001	1	0,001

**p < 0.01 CFM = Central Fat Mass; AC = Abdominal circumference n = 29

**Table 5:** Correlation matrix for CFM, Abdominal Circumference and ex-vivo Fat Mass for lean and fat rats

	CFM (g)				AC (cm)			
	
	lean n = 15		fat n = 14		lean n = 15		fat n = 14	
	
	Pearson	**Sig**.	Pearson	**Sig**.	Pearson	**Sig**.	Pearson	**Sig**.
CFM (g)	1,000	0,001	1,000	0,001				

AC (cm)	0,506 NS	0,054	0,931**	0,001	1,000	0,001	1,000	0,001

ex-vivo Fat Mass (g)	0,599*	0,018	0,734**	0,003	0,428 NS	0,112	0,583*	0,029

CFM = Central Fat Mass; AC = Abdominal Circumference; **p < 0.01; *p < 0.05; NS = not statistically significant; n = 29

## Discussion

The main result of the present study demonstrated the ability to perform an indirect determination of the visceral fat mass in rats, using the technique of DXA. In addition, this study showed that fat mass could be accurately assessed by an anthropometric technique in fat rats.

The first objective was to establish the DXA region of interest (ROI) from the whole body scan in order to determine the fat mass demonstrating the best correlation with *ex-vivo *Fat Mass obtained by weighing. In human studies, landmarks based upon the intervertebral spaces are used to determine abdominal fat [17,18]. The inter-vertebral spaces are also easily identifiable landmarks in rats. Therefore, we decided to form ROIs as rectangular boxes extending vertically from one inter-vertebral space to another. The ROI determined in our study (FM_L2_L5) was quite similar to the ROI used in humans, extending from L2 to L4 vertebrae [18].

The ROI chosen includes only a small part of peri-epydidimal adipose tissue. Indeed, the localization of this adipose tissue is lower in the abdomen. Nevertheless, correlations between the different ROIs and only perirenal or peri-epididymal adipose tissues were tested but coefficients of correlation were lower than if the two adipose tissues were combined.

Different studies [7-12] have weighed visceral fat after dissection, but no real consensus has been established to identify which fat deposit among retroperitoneal, epididymal, peri-renal and mesenteric fat tissues is more representative of visceral fat mass. In rats, dissection of mesenteric and subcutaneous fat is difficult and some errors due to dissection might occur. In contrast, removing perirenal and peri-epididymal adipose appears to constitute a reliable technique. Therefore we combined these two adipose tissues to form *ex-vivo *Fat Mass.

The strong correlation observed between CFM assessed by DXA and *ex-vivo *Fat Mass demonstrates that CFM could be a useful predictor of visceral fat mass in rats. In humans, similar results have already been observed in obese or non obese women when comparing Intra-Abdominal Fat by Magnetic Resonance Imaging and Total Fat or CFM by DXA [18,21]. In the present study, the specific analyses of these relationships were conducted in subgroups of lean and fat rats. The correlations between CFM and *ex-vivo *Fat Mass remained positive in each group, although blunted in the lean rats.

However, as DXA measures of CFM, includes abdominal fat plus peripheral subcutaneous fat, DXA values were higher than *ex-vivo *Fat Mass because it includes only the total perirenal and peri-epididymal adipose tissues. Thus, the higher values found with the DXA method can be explained by subcutaneous fat which is excluded in *ex-vivo *Fat Mass. Indeed, DXA cannot distinguish between intra-abdominal and subcutaneous fat in humans and animals [22]. Despite a small over-estimation of DXA, the strong correlation observed between CFM and *ex-vivo *Fat Mass showed that DXA could be a useful predictor of *ex-vivo *visceral fat mass in rats.

The second objective was to validate the AC measure as an anthropometric method for assessing visceral fat mass. Within the whole population of rats, a strong significant correlation was observed between AC values and both CFM by DXA and *ex-vivo *Fat Mass. These results showed that AC could be a useful tool to predict visceral fat mass. When dividing the whole population into fat and lean rats, we observed the relationships between AC, CFM and *ex-vivo *Fat Mass were maintained for the fat rats but correlations with the lean rats disappeared. The small sample of subjects resulting from the division of the whole group may affect the statistical power of relationships between variables. Despite the sample effect, these results underline the fact that when animals are not characterized by obesity, AC is no longer relevant to assess abdominal fat. These results revealed that AC is a good indicator for predicting visceral fat mass in case of obesity. To date, no other study has addressed abdominal circumference in animals. Nevertheless, different studies conducted in human subjects concluded that waist circumference is the best anthropometric predictor of intra abdominal fat thickness [5,17,18]. Similarly, the present study has shown that the AC measure could be a useful anthropometric technique for assessing *in-vivo *abdominal fat mass storage in fat rats.

## Conclusions

Both AC and CFM by DXA were good indicators for measuring abdominal fat mass storage. Coefficients of variation were found to be as low as 1.2% and 2.6% for CFM and AC, respectively. These values highlight the fact that these two measures can be used with precision and reliability. DXA and AC measures can be considered as non-invasive alternative techniques in determining visceral fat mass in rodents. These techniques have the potential to reduce the cost and number of rats required when using traditional sacrificial protocols. Moreover, abdominal circumference like other anthropometric measurements is inexpensive, quick and easy to perform. The most important outcome of the present study was that these two techniques can be used for *in-vivo *investigations, thereby allowing follow-up and longitudinal studies.

## Competing interests

The authors have no conflict of interest.

## Authors' contributions

MG has participated in the investigation as PhD student responsible of the protocol; intervention on animals, analysis and interpretation of data, drafting the manuscript.

LM has contributed to conception and design, intervention on animals, acquisition of data. ER has taken responsibilities in the housing protocol and measurements.

DC had responsibility of the design, analysis and interpretation of data and participated in drafting the manuscript, revising it critically for intellectual content and has given final approval of the version to be published.

All authors read and approved the final manuscript.
